# Association of Adjuvant Immunotherapy with Improved Survival for Stage II–III Esophageal Cancer: 4-Year National Perspective

**DOI:** 10.1245/s10434-026-19223-x

**Published:** 2026-02-20

**Authors:** Sara Sakowitz, Syed Shahyan Bakhtiyar, Yas Sanaiha, Peyman Benharash, Jane Yanagawa

**Affiliations:** 1 CORELAB, Department of Surgery, David Geffen School of Medicine, University of California Los Angeles, Los Angeles, CA USA; 2 Jonsson Comprehensive Cancer Center, David Geffen School of Medicine, University of California Los Angeles, Los Angeles, CA USA; 3 Department of Surgery, Massachusetts General Hospital, Boston, MA USA; 4 Division of Cardiothoracic Surgery, Department of Surgery, University of Utah, Salt Lake City, UT USA; 5 Department of Surgery, University of California, Los Angeles, CA USA; 6 Division of Thoracic Surgery, Department of Surgery, University of California, Los Angeles, CA USA

**Keywords:** Esophageal cancer, Esophagectomy, CheckMate 577, NCDB, Immunotherapy

## Abstract

**Background:**

The recent ground-breaking CheckMate 577 trial demonstrated that patients with esophageal cancer (EC) who underwent neoadjuvant chemoradiation and surgery followed by adjuvant immunotherapy experienced significantly improved survival. However, nationwide utilization of adjuvant immunotherapy remains unknown, as does the impact of such treatment on overall survival outside of the clinical trial setting.

**Patients and Methods:**

We identified all patients ≥ 18 years diagnosed with clinical stage II–III EC from January 2018 to December 2022 within the National Cancer Database. Only patients who underwent neoadjuvant chemoradiation and definitive esophagectomy with complete resection, found to have residual pathologic disease, were included. Patients were stratified by receipt of adjuvant immunotherapy into immunotherapy or no-immunotherapy cohorts.

**Results:**

Of 1367 patients, 342 (25%) received adjuvant immunotherapy. Utilization of immunotherapy in the adjuvant setting dramatically increased from 3% in 2018 to 50% in 2022 (*P* < 0.001). Following comprehensive risk adjustment, receipt of adjuvant immunotherapy was associated with significantly reduced mortality hazard at 1 (HR 0.25, CI 0.15–0.41) and 3 (HR 0.60, CI 0.44–0.81) years. Upon restricted mean survival time (RMST) analysis, patients who received adjuvant immunotherapy were found to experience 4.61 additional months of survival time over 3 years of follow-up (ΔRMST 4.61 months, CI 1.94–7.28).

**Conclusions:**

Utilization of adjuvant immunotherapy among patients with locally advanced residual disease following multimodal treatment has dramatically increased since the Checkmate 577 trial. In this national study, patients who received adjuvant immunotherapy experienced ~5 months of survival benefit. Yet, with such an incremental impact on survival, additional studies are needed to further optimize the multimodal perioperative treatment of patients with EC.

**Supplementary Information:**

The online version contains supplementary material available at 10.1245/s10434-026-19223-x.

Esophageal cancer (EC) remains the sixth leading cause of cancer-specific mortality.^1^ For patients with locally advanced, clinical stage II–III disease, neoadjuvant therapy followed by surgery remains the standard of care.^2,3^ However, 50–75% of such patients do not achieve a pathologic complete response (PCR),^4,5^and over one-third of these will go on to develop distant metastases.^6,7^ Unfortunately, failure to obtain PCR has been linked with a significantly inferior prognosis, with 5-year survival estimated at just 41%.^5,8^

Historically, there have been no effective adjuvant therapies to offer patients who completed neoadjuvant chemoradiotherapy and resection with remaining viable disease on final pathology. Specifically evaluating stage II–III patients who did not achieve PCR after neoadjuvant chemoradiation and surgery, the CheckMate 577 trial demonstrated that treatment with an adjuvant checkpoint inhibitor conferred significantly improved disease-free survival compared with placebo.^9^ This important trial addresses a critical need in the treatment of patients with EC; however, nationwide adoption of adjuvant immunotherapy remains unclear, as does the impact of such treatment on overall survival outside of the clinical trial setting.

Therefore, we sought to evaluate trends in the utilization of adjuvant immunotherapy, as well as survival outcomes beyond the 1-year timepoint. We hypothesized the receipt of adjuvant immunotherapy to confer superior survival at 3 years following definitive esophageal resection. We secondarily hypothesized national utilization of adjuvant immunotherapy to increase significantly across the study period.

## Patients and Methods

### Data Source

This retrospective analysis utilized the National Cancer Database (NCDB), a joint project of the American Cancer Society and the Commission on Cancer of the American College of Surgeons. The NCDB and participating hospitals are the source of the deidentified data used herein; they have not verified and are not responsible for the statistical validity of the data analysis or the conclusions derived from the authors. This study was exempted from full review by the Institutional Review Board of the University of California, Los Angeles (IRB #24-000294).

### Study Cohort

**Fig. 1:**
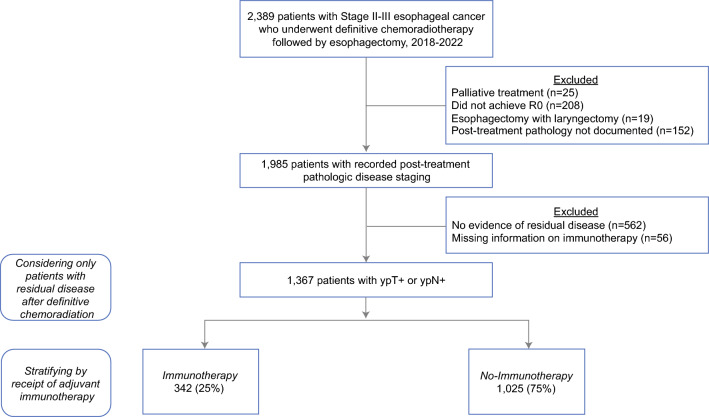
Study CONSORT diagram; records were identified within the 2018–2022 National Cancer Database; among 2389 patients with stage II–III disease who underwent definitive chemoradiotherapy followed by esophagectomy, 1367 were found to have residual disease following R0 resection, of whom 342 (25%) received adjuvant immunotherapy and 1025 (75%) did not receive such treatment

We tabulated all patients ≥ 18 years diagnosed with American Joint Committee on Cancer clinical stage II–III esophageal cancer from January 2018 to December 2022, using previously published *International Classification of Diseases for Oncology, Third Edition* histology codes.^10^ Only those who underwent neoadjuvant chemoradiation and definitive esophagectomy with a complete (R0) resection, but were found to have residual pathologic disease (ypT+ or ypN+), were considered. Patients were then stratified by receipt of adjuvant immunotherapy into immunotherapy or no-immunotherapy cohorts. Records missing key data (vital status, surgical margins) or indicating palliative treatment were excluded. We also excluded patients who received concurrent laryngectomy (Fig. 1).

### Variable Definitions

The NCDB Data Dictionary was used to characterize patient, disease, and hospital factors. Disease staging was defined according to tumor, node, metastasis 8th edition criteria. Patient burden of chronic illness was numerically represented using the modified Charlson-Deyo comorbidity index (CDI). The NCDB does not report the precise reasons why a patient did not receive immunotherapy. However, to consider the role of postoperative performance status, we defined composite major morbidity as postoperative duration of hospitalization > 20 days, readmission, or mortality within 90 days of esophagectomy.^11,12^

### Study Outcomes and Statistical Analysis

The primary endpoint of the study was overall survival at 1 year and 3 years. We secondarily considered factors linked with receipt of adjuvant immunotherapy. Continuous data are reported as means with standard deviation (SD) if normally distributed, or medians with interquartile range (IQR) otherwise. Categorical variables are represented as group frequency (%).Pearson’s chi-squared, Mann–Whitney *U*, or adjusted Wald tests were used to evaluate the significance of intergroup differences, as appropriate.

We assessed survival using Kaplan-Meier time-to-event analyses and Cox proportional hazard models. Recognizing that the immunotherapy and no-immunotherapy groups might exhibit baseline covariate variation, we applied entropy balancing to balance characteristics between groups. This method applies sample weights to improve cohort exchangeability.^13^ All model covariates were automatically selected using elastic net regularization to minimize bias and collinearity while enhancing external validity. Models ultimately adjusted for patient age, sex, CDI, race/ethnicity, primary insurance, clinical tumor stage, clinical nodal stage, tumor location, histology, year of diagnosis, hospital type, and hospital region. Restricted mean survival time (RMST) were also performed to represent the actual treatment effect of adjuvant immunotherapy. Briefly, RMST represents the average duration from death, with ΔRMST considered the absolute mean difference in survival time attributable to the treatment.^14^ We repeated our analyses using mixed-effects Cox models using gamma frailty distributions to comprehensively account for patient clustering across centers.^15^ Finally, to determine patient, disease, and hospital factors linked with receipt of adjuvant immunotherapy, we developed a multivariable, logistic regression model.

Survival was defined as the duration of time between definitive esophagectomy and point of last contact or death. Patients alive at last follow-up were censored. Model outputs are detailed as hazard ratio (HR) with 95% confidence intervals (CI). The threshold for statistical significance was set at *α* = 0.05. All statistical analyses were performed using Stata 18.0 (StataCorp LLC, College Station, TX).

## Results

### Population Characteristics and Trends

**Fig. 2:**
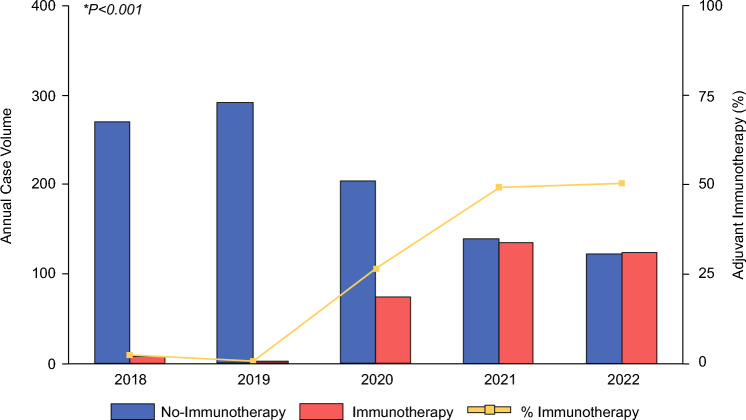
Trends in treatment approach; across the study period, utilization if adjuvant immunotherapy increased dramatically, from 3% in 2018 to 50% in 2022 (*P* for trend < 0.001)

Of 1367 patients who met study criteria, 342 (2%) received adjuvant immunotherapy and comprised the immunotherapy cohort. Utilization of immunotherapy in the adjuvant setting dramatically increased over the study period, from 3% in 2018 to 50% in 2022 (*P* for trend < 0.001, Fig. 2).

Compared with the no-immunotherapy group, the immunotherapy cohort was of incrementally younger age, but of similar sex, race, and CDI. Tumor size, clinical T and N stages, and surgical approach were also comparable between groups. Patient, disease, and hospital factors are comprehensively reported in Table 1. Notably, lower rates of composite major morbidity following esophagectomy were observed among patients who subsequently received adjuvant immunotherapy, compared with others (20 vs 32%, *P* < 0.001), reflecting differential postoperative recovery and potential treatment eligibility.

**Table 1:** Demographic, clinical, and hospital characteristics, stratified by receipt of adjuvant immunotherapy

	No-immunotherapy (*n* = 1025)	Immunotherapy (*n* = 342)	*P*-value
Age (median years [IQR])	65 [59–71]	64 [57–69]	0.007
Female	160 (16)	58 (17)	0.55
*CDI*			0.60
0–1	891 (87)	301 (88)	
2–3	134 (13)	41 (12)	
*Tumor characteristics*			
Tumor size (mean cm ± SD)	4.2 ± 2.8	4.6 ± 3.2	0.07
Squamous histology	151 (15)	42 (12)	0.26
Clinical T stage			0.23
T1	17 (2)	< 11 (NA)	
T2	245 (24)	64 (19)	
T3	745 (73)	269 (79)	
T4	11 (1)	< 11 (NA)	
*Clinical N stage*			0.86
N0	464 (45)	156 (46)	
N1	519 (51)	175 (51)	
N2	41 (4)	11 (3)	
N3	< 11 (NA)	< 11 (NA)	
*AJCC clinical stage*			0.17
II	205 (20)	57 (17)	
III	820 (80)	285 (83)	
*Pathological T stage*			0.006
T0	60 (6)	11 (3)	
Tis	24 (2)	< 11 (NA)	
T1	278 (27)	71 (21)	
T2	224 (22)	78 (23)	
T3	430 (42)	174 (51)	
T4	< 11 (NA)	< 11 (NA)	
TX	< 11 (NA)	< 11 (NA)	
*Pathological N stage*			0.12
N0	595 (58)	182 (53)	
N1	287 (28)	95 (28)	
N2	107 (10)	52 (15)	
N3	33 (3)	13 (4)	
NX	< 11 (NA)	< 11 (NA)	
*Pathological M stage*			0.13
M0	991 (98)	340 (99)	
M1	17 (2)	< 11 (NA)	
*AJCC pathological stage*			0.02
I	305 (30)	74 (24)	
II	242 (24)	96 (28)	
III	367 (36)	136 (40)	
IVA	33 (3)	17 (5)	
IVB	17 (2)	< 11 (NA)	
Unknown	61 (6)	17 (5)	
*Systemic treatment*			
Adjuvant chemotherapy	119 (12)	265 (77)	< 0.001
*Surgical approach*			0.05
Partial esophagectomy	132 (13)	58 (17)	
Total esophagectomy	129 (13)	45 (13)	
Esophagectomy with partial/total gastrectomy	655 (64)	217 (63)	
Esophagectomy, NOS	109 (11)	22 (6)	
*Race*			0.63
White	959 (94)	319 (94)	
Black	40 (4)	< 11 (NA)	
Asian/Pacific Islander	13 (1)	< 11 (NA)	
Other	< 11 (NA)	< 11 (NA)	
*Median household income percentile*			0.27
> 75th	294 (34)	114 (40)	
51st–75th	247 (29)	68 (24)	
26th–50th	201 (23)	64 (22)	
0–25th	120 (14)	39 (14)	
*Insurance coverage*			0.008
Private	392 (39)	133 (39)	
Medicare	514 (51)	150 (44)	
Medicaid	58 (6)	38 (11)	
Not insured	20 (2)	< 11 (NA)	
Other payer	32 (3)	13 (4)	
*Hospital region*			0.36
Northeast	252 (25)	85 (26)	
Midwest	258 (25)	99 (30)	
South	381 (37)	111 (33)	
West	125 (12)	38 (11)	
*Hospital type*			0.17
Academic	472 (46)	170 (51)	
Community	330 (32)	90 (27)	
Integrated network	214 (21)	73 (22)	

Reported as proportions unless otherwise noted. Statistical significance was set at *α* = 0.05

^Refers to receipt of perioperative chemotherapy and/or immunotherapy

*SD* standard deviation, *CDI* Charlson-Deyo index, *NOS* not otherwise specified

### Survival Following Resection

The median follow-up time was 1.73 years (IQR 0.89–2.94 years). On unadjusted analysis, immunotherapy demonstrated superior overall survival at 1 (91 vs 74%, log-rank *P* < 0.001), 2 (70 vs 57%, *P* < 0.001), and 3 (56 vs 46%, log-rank *P* < 0.001; Fig. 3A) years.

**Fig. 3:**
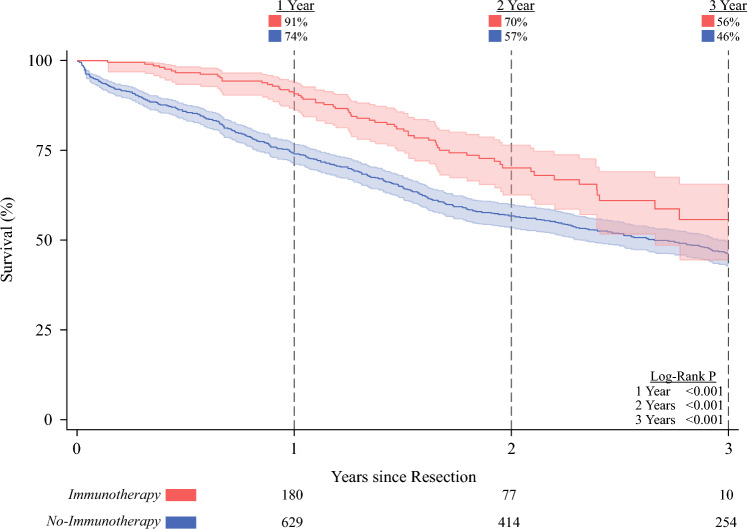
Kaplan–Meier time-to-event analysis of overall survival; patients treated with adjuvant immunotherapy demonstrated superior survival at 1 and 3 years compared with others (*P* < 0.001, log-rank test); the at-risk numbers below the figure demonstrate the actual number of patients at risk

Following application of entropy balancing and comprehensive risk adjustment, receipt of adjuvant immunotherapy was associated with significantly reduced mortality hazard at both 1 (HR 0.25, CI 0.15-0.41) and 3 (HR 0.60, CI 0.44–0.81) years (Supplementary Table S1).

Upon RMST analysis, patients who received adjuvant immunotherapy were found to experience 4.61 additional months of survival time over 3 years of follow-up, compared with those who did not undergo such treatment (ΔRMST 4.61 months, CI 1.94–7.28; Fig. 3B).

### Sensitivity Analysis Adjusting for Patient Clustering

Following multilevel modeling adjusting for patient clustering across institutions, treatment with adjuvant immunotherapy remained linked with improved survival at 1 (HR 0.25, CI 0.15–0.43) and 3 (HR 0.57, CI 0.42–0.78) years.

### Stratifying by Stage

**Fig. 4:**
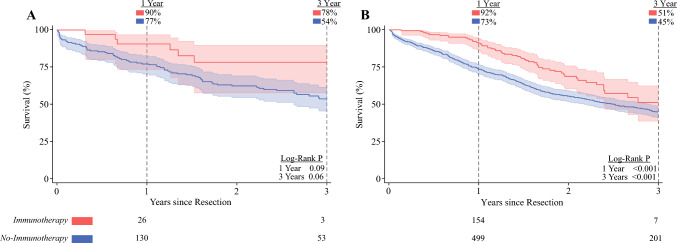
Kaplan–Meier time-to-event analyses of overall survival, stratified by clinical tumor stage; **A** among patients with clinical stage II disease, receipt of adjuvant immunotherapy was associated with statistically similar survival at 1 and 3 years, relative to others; given the absolute numerical difference in survival at 3 years, however, this subanalysis may be underpowered, and there may still be benefit of adjuvant immunotherapy for stage II patients; **B** considering those with stage III esophageal cancer, adjuvant immunotherapy treatment was linked with improved overall survival over 3 years of follow-up; the at-risk numbers below each survival curve demonstrate the actual number of patients at risk

We identified 262 patients with stage II disease, of whom 57 (22%) received adjuvant immunotherapy. Immunotherapy and no-immunotherapy demonstrated similar outcomes at 1 (90 vs 77%, log-rank *P* = 0.09) and 3 (78 vs 54%, log-rank *P* = 0.06) years. Treatment with adjuvant immunotherapy was associated with statistically similar survival over 3 years, relative to no treatment (HR 0.58, CI 0.22–1.57; Fig. 4A).

Considering the 1105 stage III patients, 285 (26%) received adjuvant immunotherapy. Immunotherapy experienced improved survival at 1 (92 vs 73%, log-rank *P* < 0.001) and 3 (51 vs 45%, log-rank *P* < 0.001) years. Notably, among this cohort, treatment with adjuvant immunotherapy was linked with significantly reduced 3-year mortality hazard (HR 0.62, CI 0.44–0.88; Fig. 4B).

### Stratifying by Residual Disease

Of the full patient cohort, 1290 (94%) had ypT+ disease and 587 (43%) ypN+ disease. Considering only those with ypT+ disease, 330 (26%) were treated with adjuvant immunotherapy. Immunotherapy demonstrated superior survival at 1 (91 vs 74%, log-rank *P* < 0.001) and 3 (55 vs 47%, log-rank *P* < 0.001) years relative to no-immunotherapy*.* Following entropy balancing and risk adjustment, adjuvant immunotherapy remained associated with superior survival at 3 years (HR 0.64, CI 0.47–0.88).

**Fig. 5:**
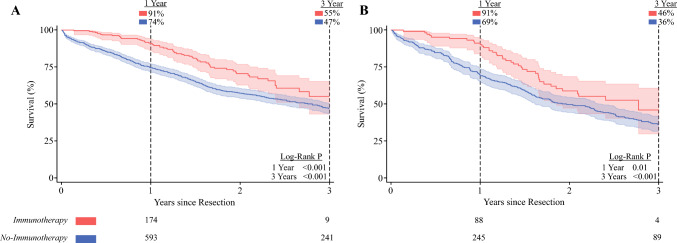
Kaplan–Meier time-to-event analyses of overall survival, stratified by patients with residual **A** ypT+ or **B** ypN+ disease; among both cohorts, receipt of adjuvant immunotherapy was associated with superior survival at 1 and 3 years; the at-risk numbers below each survival curve demonstrate the actual number of patients at risk

Meanwhile, evaluating patients with ypN+ disease, 160 (27%) received adjuvant immunotherapy. The immunotherapy cohort faced higher survival at 1 (91 vs 69%, log-rank *P* = 0.01) and 3 (46 vs 36%, log-rank *P* < 0.001) years compared with no-immunotherapy. Entropy balancing and multivariable risk adjustment revealed that adjuvant immunotherapy was again linked with improved 3-year survival (HR 0.64, CI 0.47–0.88; Fig. 5).

### Factors Linked with Adjuvant Immunotherapy

We developed a regression model to consider factors linked with likelihood of receiving adjuvant immunotherapy (*C*-statistic: 0.83). Notably, care at an academic hospital was associated with dramatically elevated odds of adjuvant immunotherapy (AOR 1.50, CI 1.07–2.12; reference: community hospital), as was more recent years of diagnosis (AOR 2.81/year, CI 2.46–3.21; reference: 2018). Meanwhile, squamous tumor histology was linked with reduced likelihood of treatment (AOR 0.56, CI 0.35–0.91; reference: adenocarcinoma), as was increasing age (AOR 0.97/year, CI 0.95–1.00). Patient sex, race, and insurance status were not linked with differential odds of adjuvant immunotherapy (Supplementary Table S2).

## Discussion

For the past decade, neoadjuvant chemoradiotherapy followed by resection has been considered the gold standard treatment for locally advanced esophageal cancer.^16,17^ While historically patients were subsequently surveilled without further treatment, the CheckMate 577 randomized controlled trial^9^ demonstrated a survival benefit of adjuvant immunotherapy among patients at high risk for disease recurrence following esophagectomy. Despite these practice-changing findings, a national analysis of adjuvant immunotherapy utilization and outcomes remains lacking. Therefore, we sought to elucidate trends in the utilization of adjuvant immunotherapy in clinical practice and survival beyond the 1-year mark.

On the basis of the findings of CheckMate 577, the American Society of Clinical Oncology updated their practice guidelines to recommend nivolumab for locally advanced patients with residual disease.^2^ Expectedly, we report a dramatic increase in the utilization of adjuvant immunotherapy for such patients across the USA, from just 3% in 2018 to 50% in 2022. While our study demonstrates a promising expansion of the utilization of immunotherapy for this cohort, our findings also highlight that ~50% of potentially eligible patients did not receive such treatment. This delta in therapeutic approach may stem, at least in part, from unequal center-level access. In fact, we found that undergoing cancer care at an academic hospital was associated with significantly elevated likelihood of receiving immunotherapy. Yet, with lower income, under-resourced patients are more likely to present to safety net institutions or community hospitals,^10,18^ and every effort must be undertaken to ensure that all patients have access to centers and care networks that provide adjuvant immunotherapy and other cutting-edge treatments. As our study only includes patients who underwent treatment through the end of year 2022, future work should verify that the number of patients receiving adjuvant immunotherapy, when indicated, improves over subsequent years. Moreover, while we could not determine the precise reasons why patients did not receive adjuvant immunotherapy, our exploratory analysis may suggest that challenges with postoperative recovery or performance status could be implicated. Given the potential survival benefit of immunotherapy, certain patients may therefore benefit from significant preoperative rehabilitation, when possible, to optimize their likelihood of proceeding and adhering to adjuvant treatment. Finally, other significant changes in treatment strategy that took place over the study period may also underlie noted trends in adjuvant immunotherapy utilization. On the basis of the findings of the ESOPEC trial, a growing cohort of patients may have received perioperative 5-FU/leucovorin/oxaliplatin/docetaxel (FLOT) and therefore would have been ineligible for immunotherapy.^17,19^ While we await a comparison of FLOT and CROSS protocols, clinicians should consider the implications of perioperative management on patient access to immunotherapy.

Notably, patients treated with adjuvant immunotherapy demonstrated significantly reduced mortality hazard over the first 3 years following esophagectomy. This association remained true after entropy balancing for patient and disease factors, including tumor histology, and adjustment for patient clustering across institutions. Altogether, our work aligns with the early results of the CheckMate 577 trial, which reported treatment with nivolumab to confer a 31% reduction in the risk of recurrence or death.^9^ However, it is equally important to note that patients treated with adjuvant immunotherapy experienced, on average, a 4.61-month increase in overall survival time over 3 years of follow-up. This statistic stands in comparison with the CheckMate 577 trial, which noted an ~11-month difference in median disease-free survival.^9^ These data similarly align with a recent single-center report that found no significant impact of immunotherapy on 1-year survival outcomes, and notable differences between a real-life versus clinical trial population.^20^ As our study reflects early populations treated with immunotherapy, it will remain paramount to continue following these results as they mature over time.

While hazard ratios favored adjuvant immunotherapy among either ypT+ or ypN+ patients, the impact on overall survival appeared to be most significant among patients with stage III disease. Most likely, there are unidentified subsets of even stage III patients, who may have a stronger response to immunotherapy than others. Indeed, current treatments for esophageal cancer largely prescribe similar treatment approaches for all disease entities by stage, without accounting for tremendous molecular heterogeneity across tumors and tumor microenvironments. In Checkmate 577, similar hazard ratios for disease recurrence or death with tumor-cell programmed death receptor ligand (PD-L1) expression either < 1% or ≥ 1% indicated that adjuvant nivolumab was similarly effective regardless of tumor-cell PD-L1 levels.^9^ However, as our collective understanding of this disease process grows, identifying relevant biomarkers will be critical to allow for regimens to be more precisely targeted to patients’ tumors, with the potential to improve PCR rates and better treat those found to have residual or recurrent disease.^21^ Furthermore, while we noted no statistically significant association between adjuvant immunotherapy and 3-year outcomes among patients with stage II disease, we underscore the presence of a large (> 20%) numerical difference in survival. Given this, there may still be considerable benefit for the subgroup of stage II patients, which the present analysis was too underpowered to detect. As the cohort of patients treated with adjuvant immunotherapy expands, we will be better able to parse the precise subgroups who would most derive benefit.

In the contemporary immunotherapy era, several important questions remain. While the CheckMate 577 trial established adjuvant immunotherapy as the standard of care for patients without PCR following chemoradiotherapy and R0 esophagectomy, it remains unclear whether those treated with perioperative chemotherapy, or who did not receive R0 resection, would similarly benefit.^22^ Additionally, the field continues to lack accurate methods or assays to accurately predict response. While immunotherapy plays a role in the neoadjuvant setting for the treatment of other cancers, its role as a neoadjuvant and perioperative treatment for EC remains to be elucidated.^23^ Finally, with increasing recognition paid to the immunotherapy side effect profile, novel efforts are needed to improve patient tolerance and quality of life. In the CheckMate 577 trial, just 43% of participants completed the full year of adjuvant treatment.^9^ Meanwhile, a single institution report found 82% of patients with locally advanced esophageal cancer terminated immunotherapy due to side effects or disease progression.^20^ Adverse events associated with immunotherapy have been suggested to be under-reported in clinical trials, with one such study finding 48% of trials did not detail patient withdrawal due to drug side effects.^24^ As immunotherapy becomes increasingly utilized in clinical practice, both comprehensive reporting of adverse effects as well as novel efforts to reduce symptom burden are urgently needed to improve patient adherence and outcomes.

Moreover, since the conclusion of our study period, treatment paradigms for resectable esophageal cancer have continued to evolve. Most notably, the landmark MATTERHORN trial demonstrated a survival benefit associated with perioperative durvalumab and FLOT chemotherapy, ultimately supporting earlier integration of immunotherapy in the neoadjuvant setting.^25^ In this context, therefore, our findings provide important real-world evidence regarding adjuvant immunotherapy use and outcomes in routine practice, in an era of transformative change in treatment strategy. As perioperative immunotherapy becomes the standard of care, our data represent an important reference point for future comparative analyses considering patient selection, treatment adherence, and outcomes across evolving multimodal regimens.

We acknowledge several important limitations of this study. First, the presence and extent of perioperative symptom burden or dysphagia were not captured. We could not identify the precise chemotherapy regimen or dose received. Our analysis relied on the accuracy of clinical staging and availability of post-therapy pathological staging data within the NCDB. Moreover, our study aligned with an intent-to-treat approach; we could identify whether patients began their adjuvant immunotherapy course, but we could not parse those who terminated treatment early. The NCDB also does not record response to neoadjuvant treatment or Mandard grade, nor does it document patient status before or after resection. PD-L1 expression levels were not reported. We could also not ascertain reasons why patients did not receive immunotherapy*.* As the NCDB does not detail disease recurrence or cause of death, our work considered overall survival. Future studies building on our analysis should consider both disease-free survival and quality of life. Finally, our median follow-up was shorter than that of CheckMate 577 (24.4 months) and therefore may not fully capture the potential benefit of immunotherapy.^25^ Perhaps the most important limitation is that the study database only includes patients treated up to the year 2022. While our study contributes a novel 4-year perspective to the literature, follow-up studies evaluating rates of utilization and more mature survival outcomes as more recent data becomes available will be valuable. Despite these limitations, we applied advanced statistical methods to a large national cohort to evaluate the utilization and impact of adjuvant immunotherapy among patients with stage II–III disease, facing high recurrence risk.

In conclusion, we report a dramatic increase in the utilization of adjuvant immunotherapy among locally advanced patients with residual disease following multimodal treatment. Treatment with adjuvant immunotherapy was associated with significantly superior survival at both 1 and 3 years following esophagectomy. While hazard ratios demonstrated the benefit of adjuvant immunotherapy, RMST analysis quantified the impact of this additional treatment at 4.61 additional months of survival time. Future work is needed to expand access to immunotherapy and optimize multimodality treatment to provide additional improvement in outcomes for patients with EC.

## Supplementary Information

Below is the link to the electronic supplementary material.

Supplementary file1 (DOCX 30 KB)
