# Supplementary material for: Association of Adjuvant Immunotherapy with Improved Survival for Stage II–III Esophageal Cancer: 4-Year National Perspective
Source: Ann Surg Oncol. 2026 Feb 20;33(6):5358–67. doi: 10.1245/s10434-026-19223-x (PMC13179238; doi:10.1245/s10434-026-19223-x)
Supplement: Supplementary file 1 — Supplementary file1 (DOCX 30 KB) [file 10434_2026_19223_MOESM1_ESM.docx]

**SUPPLEMENTARY APPENDIX**

**Association of Adjuvant Immunotherapy with Improved Survival for Stage II-III Esophageal Cancer:**

**Four-Year National Perspective**

Sara Sakowitz MD MPH MBA^1,2^, Syed Shahyan Bakhtiyar MD MBE^1,3^, Yas Sanaiha MD^1,4^, Peyman Benharash MD MS^1,4^, Jane Yanagawa MD^1,5^

^1^ CORELAB, Department of Surgery, David Geffen School of Medicine, University of California Los Angeles, Los Angeles, CA

^2^Jonsson Comprehensive Cancer Center, David Geffen School of Medicine, University of California Los Angeles, Los Angeles, CA, USA

^3^ Department of Surgery, Massachusetts General Hospital, Boston, MA

^4^ Department of Surgery, University of Colorado, Aurora, CO

^5^ Department of Surgery, University of California, Los Angeles, CA

^6^ Division of Thoracic Surgery, Department of Surgery, University of California, Los Angeles, CA

**Corresponding Authors:**

Jane Yanagawa MD jyanagawa@menet.ucla.edu

Sara Sakowitz MD MPH MBA ssakowitz@mednet.ucla.edu

**Contents**

Supplementary Table S1: Full Output of Cox Model for Overall Survival 3

Supplementary Table S2: Factors Linked with Receipt of Adjuvant Immunotherapy 4

# **Supplementary Table S1: Full Output of Cox Model for Overall Survival**

Following comprehensive risk adjustment for patient, disease, and hospital factors, receipt of adjuvant immunotherapy remained linked with a significant survival benefit over three years of follow-up. The hazard ratio with 95% confidence interval and P-value is reported for each covariate.

- CDI, Charlson-Deyo Index; NOS, Not Otherwise Specified

|  | *Hazard Ratio* | *P-Value* | *95% Confidence Interval* |
| --- | --- | --- | --- |
| Receipt of Adjuvant Immunotherapy | 0.60 | 0.001 | 0.50-0.82 |
| Age (Per Year) | 1.02 | 0.002 | 1.01-1.04 |
| Female Sex (Ref: Male) | 0.84 | 0.24 | 0.63-1.12 |
| CDI (Per Point) | 1.11 | 0.04 | 1.00-1.22 |
| T Stage (Per Incremental Increase) | 1.15 | 0.16 | 0.95-1.39 |
| N Stage (Per Incremental Increase) | 1.18 | 0.04 | 1.01-1.37 |
| *Tumor Location* |  |  |  |
| Upper/Proximal Third | Ref | - | - |
| Middle Third | 0.85 | 0.76 | 0.30-2.39 |
| Distal/Lower Third | 0.70 | 0.50 | 0.25-1.95 |
| Thoracic | 1.32 | 0.66 | 0.38-4.57 |
| NOS | 1.00 | 0.99 | 0.32-3.09 |
| *Race* |  |  |  |
| White | Ref | - | - |
| Black | 0.90 | 0.68 | 0.53-1.51 |
| Asian/Pacific Islander | 0.26 | 0.06 | 0.06-1.04 |
| Other | 0.37 | 0.17 | 0.09-1.51 |
| *Insurance* |  |  |  |
| Private | Ref | - | - |
| Medicare | 0.98 | 0.89 | 0.77-1.25 |
| Medicaid | 1.40 | 0.07 | 0.97-2.01 |
| Not Insured | 1.84 | 0.08 | 0.94-3.60 |
| Other | 1.10 | 0.72 | 0.66-1.82 |
| *Hospital Type* |  |  |  |
| Academic | Ref | - | - |
| Community | 1.10 | 0.39 | 0.89-1.35 |
| Integrated Network | 1.36 | 0.008 | 1.09-1.70 |
| Year of Diagnosis (Ref: 2018) | 1.01 | 0.77 | 0.92-1.12 |

# **Supplementary Table S2: Factors Linked with Receipt of Adjuvant Immunotherapy**

Several patient, disease, and hospital factors were found to remain associated with likelihood of receiving adjuvant immunotherapy. The hazard ratio with 95% confidence interval and P-value is reported for each covariate. The model C-statistic was 0.82.

- CDI, Charlson-Deyo Index; NOS, Not Otherwise Specified

|  | *Hazard Ratio* | *P-Value* | *95% Confidence Interval* |
| --- | --- | --- | --- |
| Age (Per Year) | 0.98 | 0.05 | 0.96-1.00 |
| Female Sex (Ref: Male) | 1.03 | 0.89 | 0.68-1.55 |
| CDI (Per Point) | 0.97 | 0.75 | 0.82-1.16 |
| T Stage (Per Incremental Increase) | 1.29 | 0.09 | 0.96-1.73 |
| N Stage (Per Incremental Increase) | 1.03 | 0.83 | 0.79-1.34 |
| Squamous Histology (Ref: Adenocarcinoma) | 0.61 | 0.04 | 0.38-0.98 |
| *Race* |  |  |  |
| White | Ref | - | - |
| Black | 0.57 | 0.19 | 0.24-1.33 |
| Asian/Pacific Islander | 1.91 | 0.28 | 0.60-6.08 |
| Other | 1.07 | 0.93 | 0.25-4.55 |
| *Insurance* |  |  |  |
| Private | Ref | - | - |
| Medicare | 0.94 | 0.75 | 0.63-1.40 |
| Medicaid | 1.55 | 0.12 | 0.89-2.70 |
| Not Insured | 0.72 | 0.59 | 0.21-2.40 |
| Other | 1.32 | 0.48 | 0.60-2.90 |
| *Hospital Type* |  |  |  |
| Academic | Ref | - | - |
| Community | 0.64 | 0.01 | 0.46-0.90 |
| Integrated Network | 0.88 | 0.51 | 0.61-1.28 |
| Year of Diagnosis (Ref: 2018) | 2.72 | <0.001 | 2.39-3.10 |
